# Patterns of distant metastasis in Chinese women according to breast cancer subtypes

**DOI:** 10.18632/oncotarget.10099

**Published:** 2016-06-16

**Authors:** San-Gang Wu, Jia-Yuan Sun, Li-Chao Yang, Li-Ying Tang, Xue Wang, Xue-Ting Chen, Gui-Hua Liu, Huan-Xin Lin, Qin Lin, Zhen-Yu He

**Affiliations:** ^1^ Department of Radiation Oncology, The First Affiliated Hospital of Xiamen University, Xiamen 361003, People's Republic of China; ^2^ Sun Yat-sen University Cancer Center, State Key Laboratory of Oncology in South China, Department of Radiation Oncology, Collaborative Innovation Center of Cancer Medicine, Guangzhou 510060, People's Republic of China; ^3^ Department of Basic Medical Science, Medical College, Xiamen University, Xiamen 361005, People's Republic of China; ^4^ Eye Institute of Xiamen University, Fujian Provincial Key Laboratory of Ophthalmology and Visual Science, Medical College, Xiamen University, Xiamen 361005, People's Republic of China; ^5^ School of Pharmaceutical Sciences, Xiamen University, Xiamen 361005, People's Republic of China

**Keywords:** breast cancer, breast cancer subtype, distant metastasis, patterns

## Abstract

To access possible relationships between breast cancer subtypes (BCS) and patterns of distant metastasis in advanced breast cancer. Breast cancer patients with distant metastasis at two academic centers from 2000-2015 were retrospectively reviewed. The breast cancer was classified into four subtypes: hormone receptor (HR) +/ human epidermal growth factor receptor 2 (HER2) − (i.e., estrogen receptor [ER] + and/or progesterone receptor [PR] +, HER2−); HR+/HER2+ (ER+ and/or PR+, HER2+), HR−/HER2+ (ER− and PR−, and HER2+); and HR−/HER2− (ER− and PR−, and HER2−). A total of 679 patients were identified. The distribution of the BCS was 39.9% (271/679), 23.7% (161/679), 16.8% (114/679), and 19.6% (133/679) in HR+/HER2−, HR+/HER2+, HR−/HER2+, and HR−/HER2−, respectively. Patients with HR+/HER2+ and HR−/HER2+ subtypes were prone to abdominal and pelvic metastasis, those with HR+/HER2− and HR+/HER2+ subtypes were prone to bone metastasis, while patients with the HR−/HER2− subtype were prone to lung/mediastinal and brain metastases. In patients with pleural, axillary and/or neck lymph node, and other distant soft tissue metastases, there was no significant difference in metastatic patterns among the BCS. There are different patterns of distant metastasis associated with different BCS. There should be a different focus in the postoperative follow-up and monitoring of breast cancer patients with different BCS.

## INTRODUCTION

Breast cancer is the most common malignancy in women worldwide. It is estimated that there will be about 232,000 new cases of breast cancer diagnosed in the United States in 2015 [1]. In China, there is also a rapid growth trend in breast cancer, and cancer prevalence estimates for 5 years are 1.02 million women with breast cancer [2]. Although great progress has been made in the comprehensive treatment of breast cancer, 20%-30% of patients will still develop distant metastases [3–5]. Bone, lung, liver, and brain are the most common metastatic sites of breast cancer [6], but there is a difference in the survival of patients for different metastatic sites [7, 8].

Currently, common risk factors for distant metastasis of breast cancer include tumor size, nodal stage, histological grade, estrogen receptor (ER), progesterone receptor (PR), human epidermal growth factor receptor 2 (HER2), and others [9–11]. Traditional tumor-node-metastasis (TNM)-staging may predict the risk of breast cancer metastasis and death, but the predictive value for specific sites of metastasis is poor. Breast cancer, a heterogeneous disease composed of distinct biological subtypes, can be divided into four simple subtypes based on ER, PR and HER2 status: hormone receptor (HR)+/HER2−, HR+/HER2+, HR−/HER+, and HR−/HER2− [12–14]. The breast cancer subtypes (BCS) are increasingly recognized as predictive factors for disease control and response to adjuvant therapies including chemotherapy, radiotherapy and targeted therapy [15–17]. However, data are limited and conflict concerning differences in specific sites of distant metastasis among the various BCS [18–21]. In this study, we sought to access the possible relationships between BCS and patterns of distant metastasis in advanced breast cancer patients from two cancer centers to aid in the development of personalized programs of surveillance.

## RESULTS

### Patient characteristics

Six hundred and seventy-nine patients were identified, 493 (72.6%) from Sun Yat-sen University Cancer Center (SYSUCC) and 186 (27.4%) from the First Affiliated Hospital of Xiamen University (Xiamen Cancer Center, XMCC). Table 1 shows clinicopathological data of the patients. The median age was 46.8 years (range 23-87) when breast cancer was diagnosed, 64.5% (438/679) of patients were premenopausal. Five hundred and twenty-nine patients (77.9%) were in Tumor (T)1-T2 stage, and 371 patients (54.6%) were in Node (N)2-N3 stage. In patients with distant metastasis, HR+/HER2−, HR+/HER2+, HR−/HER2+ and HR−/HER2− BCS accounted for 39.9% (271/679), 23.7% (161/679), 16.8% (114/679), and 19.6% (133/679), respectively. Nodal stage was significantly different among the four BCS (P = 0.045) (Table 2). No significant differences in age, menopausal status, tumor size, and histotype were found among the four BCSs.

**Table 1:** Summary of characteristics in 679 patients enrolled in this study

Characteristic	n	SYSUCC	XMCC
Age (median, years)	46.8 ± 10.8	46.1 ± 10.7	48.5 ± 10.9
Menopausal status			
Premenopausal	438	322	116
Postmenopausal	241	171	70
Tumor size			
T1	161	115	46
T2	368	264	104
T3	105	76	29
T4	45	38	7
Nodal stage			
N0	171	130	41
N1	137	93	44
N2	167	122	45
N3	204	148	56
Histotype			
Invasive ductal carcinoma	638	471	167
Other	41	22	19
Ki-67 (n = 310)			
≤25% positive	152	130	22
>25% positive	158	89	69
Breast cancer subtype			
HR+/HER2−	271	193	78
HR+/HER2+	161	111	50
HR−/HER2+	114	91	23
HR−/HER2−	133	98	35
Site of distant metastasis (n = 1025)			
Abdomen/pelvis	221	172	49
Lung/mediastinum	248	179	69
Pleura	62	34	28
Bone	308	201	107
Axillary and/or neck lymph nodes	69	42	27
Brain	78	47	31
Other distant soft tissue	39	24	15

SYSUCC, Sun Yat-sen University Cancer Center; XMCC, Xiamen Cancer Center; T, tumor; N, node; HR, hormone receptor; HER2, human epidermal growth factor receptor 2.

**Table 2:** Clinicopathological characteristics according to breast cancer subtype

Characteristic	HR+/HER2−	HR+/HER2+0	HR−/HER2+	HR−/HER2−	*P value*
Age (median, years)	46.6 ± 11.3	46.0 ± 10.9	48.4 ± 10.7	46.8 ± 9.7	0.345
Menopausal status					
Premenopausal	181	109	62	86	0.092
Postmenopausal	90	52	52	47	
Tumor size					
T1	66	31	28	36	0.058
T2	152	88	61	67	
T3	28	35	19	23	
T4	25	7	6	7	
Nodal stage					
N0	66	36	25	44	0.045
N1	67	26	19	25	
N2	69	39	34	25	
N3	69	60	36	39	
Histotype					
Invasive ductal carcinoma	251	151	111	125	0.355
Other	20	10	3	8	

T, tumor; N, node; HR, hormone receptor; HER2, human epidermal growth factor receptor 2.

### Distant metastasis of patients

**Figure 1:**
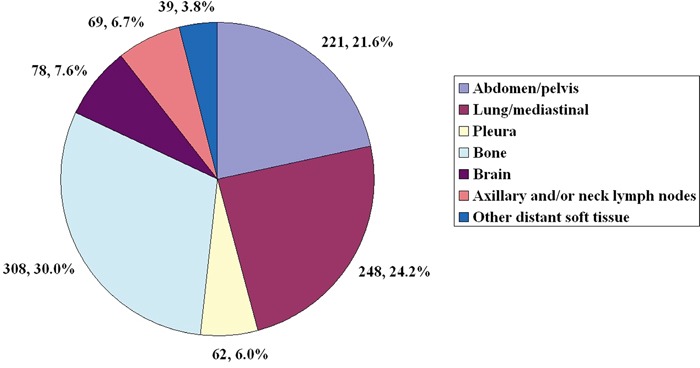
The frequencies of the sites of distant metastasis

The median follow-up period among patients diagnosed with metastatic breast cancer was 26.7 months. The 1-year, 2-year, and 3-year overall survival (OS) was 74.3%, 49.3% and 34.5%, respectively. The median distant metastasis time of HR+/HER2−, HR+/HER2+, HR−/HER2+ and HR−/HER2− was 41.0 ± 26.9 months, 32.3 ± 27.6 months, 22.8 ± 15.8 months and 26.9 ± 20.6 months, respectively (P < 0.001). Of the 679 patients, there were 1025 sites of distant metastases were definitely identified (Figure 1); 445 patients had a solitary metastasis and 234 patients had multiple metastases. Common sites of metastasis included bone (30.0%, 308/1025), lung/mediastinum (24.2%, 248/1025), abdomen/pelvis (21.6%, 221/1025), brain (7.6%, 78/1025), axillary and/or neck lymph nodes (6.7%, 69/1025), pleura (6.0%, 62/1025), and other distant soft tissue (3.8%, 39/1025). There was no significant correlation between BCS and the number of distant organ metastases (P = 0.674). Univariate and multivariate analysis showed that nodal stage was a risk factor affecting lung/mediastinal, and axillary and/or neck lymph node metastasis (P < 0.05), while histotype was a risk factor affecting pleural metastasis (P < 0.05). Age, menopausal status, tumor size and Ki-67 level did not affect the patterns of distant metastasis.

### Association of breast cancer subtypes with the sites of distant relapse

**Figure 2:**
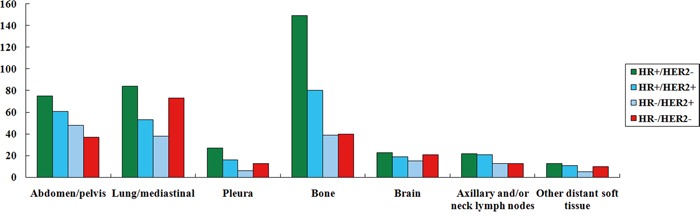
The frequencies of the sites of distant metastasis by breast cancer subtypes

Univariate and multivariate analysis showed that HR+/HER2+ and HR−/HER2+ subtype patients had a higher probability of abdominal/pelvic metastasis compared to HR+/HER2− subtype patients, while the probability of abdominal/pelvic metastasis of the HR−/HER2+ subtype was higher than that of the HR−/HER2− subtype. Multivariate analysis showed that the probability of abdominal/pelvic metastasis of the HR+/HER2+ subtype was also higher than that of the HR−/HER2− subtype (Table 3; Figure 2).

**Table 3:** Specific breast cancer subtypes associated with the sites of distant metastasis

Site of distant metastasis/subtype	Univariate			Multivariate		
	OR	95% CI	*P value*	OR	95% CI	*P value*
Abdomen/pelvis						
HR+/HER2+ vs. HR+/HER2−	1.594	1.053-2.414	0.028*	1.665	1.096-2.530	0.017*
HR−/HER2+ vs. HR+/HER2−	1.901	1.203-3.002	0.006*	1.971	1.244-3.124	0.004*
HR−/HER2− vs. HR+/HER2−	1.007	0.634-1.601	0.976	0.933	0.624-1.581	0.977
HR−/HER2+ vs. HR+/HER2+	1.192	0.731-1.945	0.481	1.184	0.724-1.936	0.501
HR−/HER2− vs. HR+/HER2+	0.632	0.385-1.037	0.069	0.596	0.662-0.984	0.043*
HR−/HER2+ vs. HR−/HER2−	1.887	1.109-3.209	0.019*	1.964	1.147-3.361	0.014*
Lung/mediastinum						
HR+/HER2+ vs. HR+/HER2−	1.092	0.720-1.659	0.678	1.138	0.747-1.733	0.548
HR−/HER2+ vs. HR+/HER2−	1.113	0.698-1.775	0.653	1.150	0.719-1.838	0.560
HR−/HER2− vs. HR+/HER2−	2.709	1.766-4.154	< 0.001*	2.697	1.755-4145	< 0.001*
HR−/HER2+ vs. HR+/HER2+	1.019	0.612-1.696	0.943	1.010	0.606-1.686	0.968
HR−/HER2− vs. HR+/HER2+	2.479	1.543-3.983	< 0.001*	2.370	1.471-3.820	< 0.001*
HR−/HER2+ vs. HR−/HER2−	0.411	0.245-0.690	0.001*	0.425	0.252-0.717	0.001*
Pleura						
HR+/HER2+ vs. HR+/HER2−	0.997	0.520-1.913	0.993	1.029	0.533-1.987	0.932
HR−/HER2+ vs. HR+/HER2−	0.502	0.201-1.251	0.139	0.552	0.220-1.387	0.206
HR−/HER2− vs. HR+/HER2−	0.979	0.488-1.965	0.952	0.991	0.491-2.000	0.981
HR−/HER2+ vs. HR+/HER2+	0.503	0.191-1.329	0.166	0.537	0.201-1.429	0.213
HR−/HER2− vs. HR+/HER2+	0.982	0.454-2.122	0.963	0.963	0.442-2.101	0.925
HR−/HER2+ vs. HR−/HER2−	0.513	0.188-1.396	0.191	0.557	0.203-1.527	0.256
Bone						
HR+/HER2+ vs. HR+/HER2−	0.809	0.547-1.195	0.287	0.809	0.547-1.195	0.287
HR−/HER2+ vs. HR+/HER2−	0.426	0.270-0.671	< 0.001*	0.426	0.270-0.671	< 0.001*
HR−/HER2− vs. HR+/HER2−	0.352	0.227-0.547	< 0.001*	0.352	0.227-0.547	< 0.001*
HR−/HER2+ vs. HR+/HER2+	0.527	0.321-0.864	0.011*	0.527	0.321-0.864	0.011*
HR−/HER2− vs. HR+/HER2+	0.435	0.269-0.706	0.001*	0.435	0.269-0.706	0.001*
HR−/HER2+ vs. HR−/HER2−	1.209	0.708-2.066	0.487	1.251	0.729-2.147	0.417
Brain						
HR+/HER2+ vs. HR+/HER2−	1.443	0.759-2.741	0.263	1.462	0.768-2.783	0.248
HR−/HER2+ vs. HR+/HER2−	1.634	0.819-3.260	0.164	1.690	0.843-3.388	0.140
HR−/HER2− vs. HR+/HER2−	2.022	1.074-3.805	0.029*	2.054	1.089-3.874	0.026*
HR−/HER2+ vs. HR+/HER2+	1.132	0.549-2.336	0.736	1.156	0.558-2.396	0.697
HR−/HER2− vs. HR+/HER2+	1.401	0.718-2.734	0.322	1.405	0.719-2.747	0.320
HR−/HER2+ vs. HR−/HER2−	0.808	0.395-1.653	0.559	0.833	0.404-1.717	0.620
Axillary and/or neck lymph nodes						
HR+/HER2+ vs. HR+/HER2−	1.698	0.902-3.197	0.101	1.588	0.836-3.016	0.158
HR−/HER2+ vs. HR+/HER2−	1.457	0.707-3.004	0.308	1.420	0.680-2.963	0.350
HR−/HER2− vs. HR+/HER2−	1.226	0.597-2.518	0.579	1.262	0.611-2.608	0.529
HR−/HER2+ vs. HR+/HER2+	0.858	0.410-1.794	0.684	0.894	0.422-1.892	0.770
HR−/HER2− vs. HR+/HER2+	0.722	0.347-1.504	0.384	0.795	0.378-1.671	0.545
HR−/HER2+ vs. HR−/HER2−	1.188	0.527-2.679	0.678	1.128	0.495-2.567	0.775
Other distant soft tissue						
HR+/HER2+ vs. HR+/HER2−	1.455	0.636-3.330	0.374	1.410	0.613-3.242	0.419
HR−/HER2+ vs. HR+/HER2−	0.910	0.317-2.616	0.862	0.921	0.318-2.665	0.880
HR−/HER2− vs. HR+/HER2−	1.614	0.688-3.782	0.271	1.664	0.707-3.915	0.243
HR−/HER2+ vs. HR+/HER2+	0.626	0.211-1.852	0.397	0.654	0.220-1.942	0.444
HR−/HER2− vs. HR+/HER2+	1.109	0.456-2.697	0.820	1.181	0.482-2.891	0.716
HR−/HER2+ vs. HR−/HER2−	0.564	0.187-1.702	0.310	0.564	0.186-1.712	0.312

* Indicates a significant difference at P < 0.05.

HR, hormone receptor; HER2, human epidermal growth factor receptor 2; OR, odds ratio; CI, confidence interval.

Univariate and multivariate analysis showed that HR−/HER2− had a significantly higher probability of lung/mediastinal metastasis than the other three subtypes. There were no significant differences in the probability of lung/mediastinal metastases among the other three subtypes (Table 3; Figure 2).

In terms of bone metastases, univariate and multivariate analysis showed that the probability of bone metastasis of HR+/HER2− and HR+/HER2+ subtypes was significantly higher than that of the HR−/HER2+ and HR−/HER2− subtypes, while there was no significant difference in the probability of bone metastasis between HR+/HER2− and HR+/HER2+ (Table 3; Figure 2).

The probability of brain metastasis of HR−/HER2− was significantly higher than that of the HR+/HER2− subtype, but there were no significant differences in brain metastasis among the other subtypes (Table 3; Figure 2).

## DISCUSSION

In this study, we investigated the relationships between BCS and distant metastasis sites of breast cancer. The various BCSs had site-specific metastasis patterns, patients with HR+/HER2+ and HR−/HER2+ subtypes were prone to abdominal/pelvic metastasis, patients with HR+/HER2− and HR+/HER2+ subtypes were prone to bone metastasis, while patients with the HR−/HER2− subtype were prone to lung/mediastinal and brain metastases.

Breast cancer subtype is an important factor affecting the survival of breast cancer patients irrespective of distant metastasis. In general, the survival of HR+ patients is superior to that of HER2+ and HR−/HER2− (also called triple-negative breast cancer [TNBC]) patients [15–17]. Park et al. found that BCS did not affect the OS of patients with early recurrence (distant metastases within 24 months after surgery; P = 0.08), but for patients with distant metastases ≥24 months after surgery, BCS significantly affected the OS, and the survival of HR+/HER2− and HR−/HER2+ patients was superior to that of the HR+/HER2+ and HR−/HER2− groups (P < 0.001) [18]. Considering the St Gallen molecular subtypes (2013), Gerratana et al. found that the median survival time for luminal HER2+ was 56.7 months, luminal A 45.3 months, luminal B 31.1 months, non-luminal HER2+ 21.5 months, and TNBC 9.3 months (P < 0.0001) [19]. Based on our results and related studies, BCS has both prognostic value for newly diagnosed and advanced breast cancer patients, and can also predict the patterns of distant metastases.

There may be organ-specific metastases associated with different BCSs, which supports the hypothesis that breast cancer is a systemic disease with heterogeneous characteristics. Bone metastasis is the most common metastasis of breast cancer, but the predictive value of BCS for bone metastasis is still controversial. Previous studies have tended to suggest that HR+ patients are more prone to bone metastases [19, 22–24]. Our results also showed that the probability of bone metastasis in patients with HR+ subtypes (HR+/HER2− and HR+/HER2+) was significantly higher than that for patients with HR− subtypes (HR−/HER2− and HR−/HER2+). However, two studies of the Korean population did not find a significant correlation between BCS and bone metastasis [18, 20]. In a study of the Chinese population, the probability of bone metastasis in patients with a HER2+ subtype was lower than in TNBC (P = 0.048), but there was only borderline significance compared with HR+/HER2− patients (P = 0.058) [25].

Our study found that breast cancer with the HER2+ subtype (HR+/HER2+ and HR−/HER2+) is more prone to abdominal and pelvic metastases than the other BCS, and most of patients with abdominal and pelvic metastases were liver metastases. The study of Kennecke et al. found that the probability of liver metastasis in patients with luminal A (ER+ and/or PR+ and Ki-67 <14%), luminal B (ER+ and/or PR+ and Ki-67 ≥14%), HR+/HER2+, HR−/HER2+ and TNBC subtypes was 7.9%, 13.8%, 21.3%, 23.3% and 10.7%, respectively [21]. The study of Park et al. did not find differences in liver metastasis between different subtypes [18], but there were only 18 patients with liver metastases in Park's study, whereas the number of patients with abdominal and pelvic metastasis in our study was 221, and there were 435 patients with liver metastasis in the study of Kennecke et al. [21]. The large differences in sample size may be the main reason leading to different results. In our study and the research of Kennecke et al., most of the patients did not take trastuzumab treatment; therefore, it is not yet clear whether anti-HER2 therapy may affect the patterns of distant metastases. However, in the study of Olson et al., 113 HER2+ patients who received trastuzumab-based therapy diagnosed with distant metastases during the follow-up period, and 41% of these patients had liver metastases [26], suggesting that anti-HER2 therapy may not affect the patterns of distant metastases.

Because of the lack of appropriate therapeutic targets, patients with TNBC exhibit a poor prognosis due to occurred early distant metastasis [6]. Our results showed that the median distant metastasis time of TNBC was significantly earlier than HR+ breast cancer, while the probability of lung metastasis in patients with TNBC was significantly higher than for the other three subtypes. The research of Soni et al. also found that the probability of lung metastasis of TNBC was significantly higher than that of the HR+/HER2− and HR+/HER2+ subtypes, but was not different from HR−/HER2+ [27]. In advanced TNBC, the probability of lung metastasis can reach to 40% compared with only 20% in non-TNBC [6]. There was an overexpression of epidermal growth factor receptor (EGFR) in > 50% of patients with TNBC [28, 29], and it was found from a tumor microarray study that patients with significantly elevated expression of EGFR were more prone to lung metastasis [22]. It has found that the EGFR inhibitors erlotinib could prevent development of lung metastases in a spontaneous lung metastasis breast cancer mouse model [30]. In addition, patients with high expression of EGFR were more prone to brain metastases [31], which is consistent with the high probability of brain metastases of TNBC in other studies [21, 32] as well as ours.

There are several limitations of the present study. First, there is an inherent bias that exists in any retrospective study. However, a major strength of the study is that the large number of patients with distant metastases in this cohort allowed clear demonstration of the distant metastasis patterns according to BCS. Second, the time span of the patients included is large, while the adjuvant treatment of breast cancer has made rapid progress in recent years; therefore the systemic therapy guidelines during the era of this study are not representative of current practice guidelines. But whether these will affect the patterns of breast cancer metastasis is still unclear. In addition, Ki-67 is an important marker for the molecular subtypes, but Ki-67 data was not available for >50% of the patients in our study. Therefore, Ki-67 was not used as a marker for the BCS in our study. However, Kennecke et al. found that the distant metastasis rate in various organs in luminal B (ER+ and/or PR+ and Ki-67 ≥14%) patients was higher than for luminal A (ER+ and/or PR+ and Ki-67 <14%) [21], indicating that Ki-67 had a potential impact on the metastasis patterns.

In conclusion, or results showed that the BCS based on ER, PR and HER2 status have different patterns of distant metastasis. There should be a different focus in postoperative follow-up and monitoring for breast cancer patients with various BCS, and there should be further exploration of the individualized treatment for different BCS to reduce the risk of specific sites of distant metastases.

## MATERIALS AND METHODS

### Patients

A retrospective analysis was conducted of breast cancer patients who underwent surgery in SYSUCC and the First Affiliated Hospital of Xiamen University (Xiamen Cancer Center, XMCC) from December 2000 to April 2015. Patients in the study were met the following criteria: 1) female, unilateral invasive breast cancer without distant metastasis in the initial diagnosis; 2) received mastectomy or breast-conserving surgery and axillary lymph node dissection; 3) sites of metastases were definitely identified during follow-up; 4) complete data on the following: age, menopausal status, tumor size, nodal status, histotype, and ER, PR as well as HER2 status. We excluded patients with primary cancer before the diagnosis of breast cancer and second cancer after breast cancer. The study was approved by the Ethics Committees of the SYSUCC and XMCC.

### Clinicopathological factors

Age, menstrual status, T stage, N stage, histotype and BCS were used to evaluate the patterns of distant metastasis. HR positivity was defined as ≥1% positive cells in ER or PR immunohistochemistry. HER2 positivity was defined as an immunohistochemical grade of 3+ (uniform and intensity membrane staining of > 30% of invasive tumor cells), or (after 2003 only) of 2+ determined by dual-probe fluorescence *in situ* hybridization. The cut-off point of higher Ki-67 expression was defined as 25% based on our previous studies [33]. Since Ki-67 data was missing for many patients, the BCS was not defined according to the St Gallen International Expert Consensus [34]. Instead, we defined four-major intrinsic BCS [11–13]: HR+/HER2− (ER+ and/or PR+, HER2-), HR+/HER2+ (ER+ and/or PR+, HER2+), HR−/HER2+ (ER−, PR− and HER2+) and HR−/HER2− (ER−, PR− and HER2−).

### Sites of distant metastasis

The synchronous metastatic sites of breast cancer were classified into seven areas in previous study, including abdomen/pelvis (liver, adrenal gland, lymph nodes, and other abdomino-pelvic organs); lung/mediastinum (lung or pulmonary lymphangitic spread); bone (skeletal system); pleura (pleura and/or pleural effusion and/or pericardial effusion); brain; axillary and/or neck lymph nodes; and other distant soft tissue [35].

### Statistical analysis

All data were analyzed using the SPSS statistical software package (version 21.0; IBM Corporation, Armonk, NY, USA). The χ^2^ and Fisher's exact probability tests were used to analyze the differences between qualitative data. The continuous variables were compared using Student's t-test. The association of patient characteristics factors and patterns of distant metastasis was modeled with univariate and multivariable logistic regression analysis. Predictive factors for distant metastasis were determined by multivariable logistic regression analysis, in which factors that were statistically significant in univariate analysis were entered into the multivariable logistic regression analysis. A P-value < 0.05 was considered significant in all analyzes.
